# Silencing of Angiopoietin-Like Protein 4 (Angptl4) Decreases Inflammation, Extracellular Matrix Degradation, and Apoptosis in Osteoarthritis via the Sirtuin 1/NF-*κ*B Pathway

**DOI:** 10.1155/2022/1135827

**Published:** 2022-08-27

**Authors:** Chao Jia, Xiucui Li, Jun Pan, Haiwei Ma, Dengying Wu, Hongwei Lu, Wei Wang, Xutong Zhang, Xianhong Yi

**Affiliations:** ^1^Department of Orthopaedics, The Second Affiliated Hospital and Yuying Children's Hospital of Wenzhou Medical University, Wenzhou, Zhejiang 325027, China; ^2^The Second School of Medicine, Wenzhou Medical University, Wenzhou, 325027 Zhejiang Province, China; ^3^Bone Research Institute, The Key Orthopaedic Laboratory of Zhejiang Province, Wenzhou, China; ^4^Department of Neonatology, The Second Affiliated Hospital and Yuying Children's Hospital of Wenzhou Medical University, Wenzhou, 325027 Zhejiang Province, China; ^5^Department of Anesthesiology, The Second Affiliated Hospital and Yuying Children's Hospital of Wenzhou Medical University, Wenzhou, Zhejiang 325027, China

## Abstract

Osteoarthritis (OA) is a frequently observed condition in aged people. OA cartilage is characterized by chondrocyte apoptosis, chondrocyte inflammation, and hyperactive catabolism of extracellular matrix. However, the specific molecular mechanisms remain unclear. Recent data has shown that Angptl4, a multifunctional cytokine, is involved in the regulation of inflammatory and apoptosis responses in different tissues. This study is aimed at defining the role of Angptl4 in the development of OA. We employed X-ray analysis, safranin O-fast green (S-O) staining, and hematoxylin staining to evaluate histomorphological characteristics in the knee joint of mice. Real-time quantitative polymerase chain reaction, Western blot assays, immunofluorescence staining, and enzyme-linked immunosorbent assays (ELISA) were performed to analyze the changes in gene and protein expression. Mechanically, our data demonstrated that Angptl4 knockdown improved the degradation of extracellular matrix and reduced TNF-*α*-mediated chondrocyte inflammation and apoptosis by suppressing sirtuin 1/NF-*κ*B signaling pathway. In addition, animal studies showed that the suppression of Angptl4 expression might alleviate OA development. In conclusion, our findings revealed the underlying mechanisms of Angptl4 regulation in chondrocytes and its potential value in the treatment of OA.

## 1. Introduction

Osteoarthritis (OA) is a chronic, degenerative, and debilitating form of arthritis, which is the main cause for pain and disability among old people [1]. OA exerts a heavy burden on society, because it does not only impact the patient's quality of life and performance but also it is expensive to manage [2]. Risk factors such as joint trauma, aging, sex, genetic predisposition, and obesity have been shown to induce joint destruction [3]. However, data on pathological mechanisms involved in OA progression remain scant [4]. Current treatment options for OA focus on the alleviation of symptoms, which often recur [5]. Understanding OA pathogenesis is pivotal in the development of novel therapeutic options for OA.

Chondrocytes are a unique articular cartilage cell type that generate extracellular matrix (ECM) molecules such as proteoglycans and collagen, which are the major articular cartilage components [6, 7]. However, during OA, proinflammatory factors such as interleukin- (IL-) 1*β* and tumor necrosis factor-*α* (TNF-*α*) fuel the degeneration of chondrocytes by enhancing production of inflammatory cytokines and matrix metalloproteinases (MMPs) [8]. Previous studies have demonstrated that ECM decomposition, inflammation, and apoptosis affect vital pathways that lead to OA [9]. Nonetheless, factors and underlying mechanisms that regulate ECM apoptosis, inflammation, and metabolism remain unclear.

In addition, recent studies have suggested that Sirtuin 1 slows the genesis and development of inflammation in human chondrocytes, which is achieved by the deacetylation of nuclear factor kappa B (NF-*κ*B), thus suppressing expression of various inflammation-related genes [10, 11]. SIRTUIN 1 is a type of Sir2 homolog in mammals that belongs to class III histone deacetylase and has been shown to deacetylates various nonhistone substrates and histones [12]. Besides, SIRTUIN 1 has been extensively reported to slow key physiological and metabolic processes, such as aging, apoptosis, stress tolerance, and metabolism [13, 14]. In addition, a previous study demonstrated that there is a need to prevent excessive activation of the NF-*κ*B signaling pathway when treating OA [15]. Therefore, SIRTUIN 1/NF-*κ*B is a crucial signaling pathway that would modulate normal development and pathological destruction of the cartilage [10].

There is growing evidence that angiopoietin-like protein (ANGPTL) is associated with apoptosis, inflammation, tumor development, and metabolism [16–19]. Previous studies have illustrated that increased expression of Angptl4 in synovial fibroblasts within arthritic tissues [20] and angiopoietin family proteins contributes to the pathogenesis of OA [21, 22]. Angiopoietin-like proteins 4 (Angptl4), a cytokine, has been shown to be a potential therapeutic target in various diseases [23]. Angptl4 is produced in adipose tissues and liver and participates in metabolism of lipids and glucose [24]. There are reports that Angptl4 stimulates osteoclast resorption activity in vitro; it may therefore mediate osteolytic erosion of bone and cartilage in RA [25]. Angptl4 was recently shown to be associated with a variety of diseases, such as diabetes [26], pancreatitis [27], coronary artery disease (CAD) [28], and some musculoskeletal diseases including disc degeneration, rheumatoid arthritis, and osteoporosis [29]. Besides, Angptl4 promotes osteoclast-mediated bone resorption, cartilage degradation, angiogenesis, and vascular permeability, as well as tumor cell growth and metastasis [30].

Previous studies have shown that chronic inflammation with reduced oxygen tension may upregulate Angptl4 expression in diseased joints and further induce cartilage matrix degradation [31]. And Angptl4 promotes resorption of the mature cartilage matrix by inhibited gene expression of aggrecan and type II collagen, while it increased that of matrix metallopeptidases [32]. However, the mechanisms by which the Angptl4 causes the adverse effects of osteoarthritis has not been fully elucidated. Besides, although Angptl4 plays a role in inflammation and apoptosis [33], its exact function and mechanisms are context-dependent and largely undetermined, especially in OA. Thus, we investigated the exact role of Angptl4 in TNF-*α*-induced OA chondrocytes *in vitro* and in DMM-induced OA *in vivo*. Several studies have shown that Angptl4 has more receptor-mediated and intracellular activities, including NF-*κ*B-regulated inflammation, and interaction with SIRTUIN 1 and ROS [34, 35]. A previous study also showed that silencing Angptl4 protected the inflammatory response and apoptosis in LPS-induced acute lung injury by regulating the SIRTUIN 1/NF-*κ*B signaling pathway [16]. Based on the previous studies, we hypothesized that silencing Angptl4 could improve OA, which might be mediated by SIRTUIN 1/NF-*κ*B signaling pathway.

This study analyzed the expression of Angptl4 in articular cartilage cells from normal and OA samples. Thereafter, we performed overexpression or knockdown of Angptl4 and evaluated its function in chondrocyte apoptosis, ECM metabolism, and inflammation. In addition, Angptl4 knockdown lentivirus was intra-articularly injected to evaluate its efficacy in suppressing OA progression. Our analysis showed that Angptl4 enhances chondrocyte apoptosis and inflammation and induces imbalance in ECM metabolism by regulating the SIRTUIN 1/NF-*κ*B signal pathway. Thus, Angptl4 suppression might be a novel treatment strategy for OA.

## 2. Results

### 2.1. Angptl4 is Upregulated in Human and Mouse Knee Articular Cartilage

**Figure 1:**
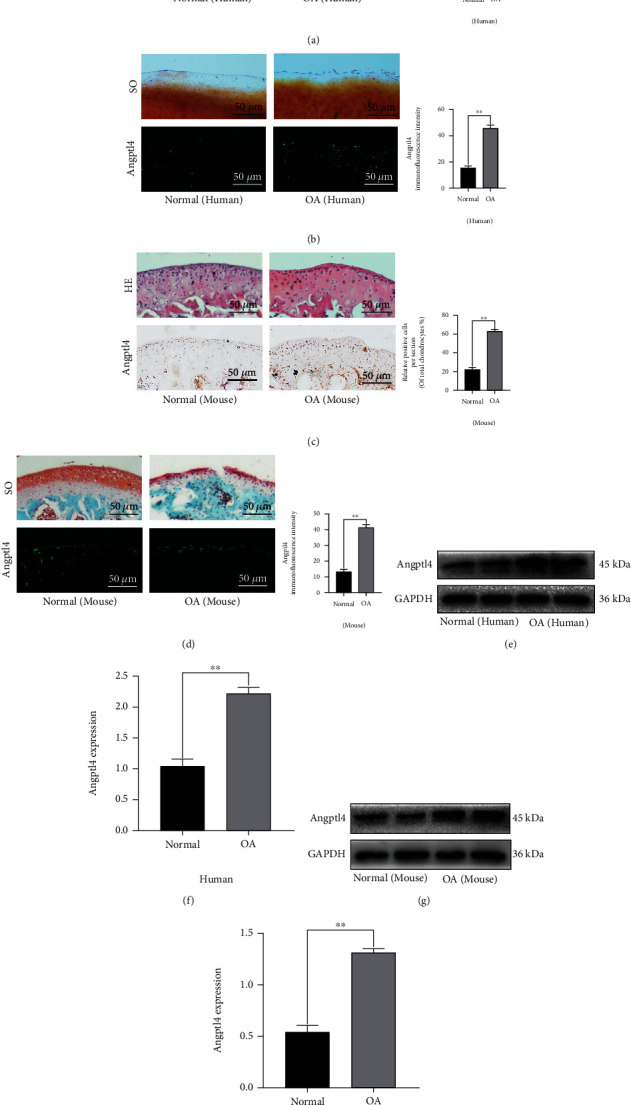
The expression of Angptl4 was increased in osteoarthritic human and mouse articular cartilage. (a, b) Representative H&E staining, Safranin O staining, immunofluorescence staining, and immunohistochemistry staining images of human articular cartilage from healthy control and OA patients (bar: 50 *μ*m). (c, d) Representative H&E staining, Safranin O staining, immunofluorescence staining, and immunohistochemical staining images of mouse knee articular cartilage from the sham and DMM models (bar: 50 *μ*m). (e, f) The protein expression of Angptl4 in chondrocytes obtained from normal and degenerated human specimens. (g, h) The protein expression of STING in chondrocytes collected from normal group and DMM (degenerated) groups. All data are presented as the mean ± SD (*n* = 5); ^∗∗^*P* < 0.01.

To determine the role of Angptl4 in OA pathogenesis, we determined the expression of Angptl4 in healthy and osteoarthritic tissues in humans and mice. Immunofluorescence and immunohistochemistry data showed that Angptl4 was highly expressed in OA articular cartilage compared to unaffected counterparts (Figures 1(a) and 1(b)). Besides, compared with the control group, there was remarkable upregulation of Angptl4 in OA articular cartilage, which was associated with destabilization of the medial meniscus (DMM) (Figures 1(c) and 1(d)). In addition, we analyzed the chondrocytes in the knee joint cartilage of normal individuals and patients with OA using Western blot assays. The data showed that the expression of Angptl4 was higher in chondrocytes derived from patients with OA than in those derived from healthy patients (Figures 1(e) and 1(f)). In addition, our data showed that Angptl4 expression was markedly increased in DMM-mediated OA mice than in the control group (Figures 1(g) and 1(h)).

### 2.2. Treatment with TNF-*α* Promotes the Expression of Angptl4 in Chondrocytes

**Figure 2:**
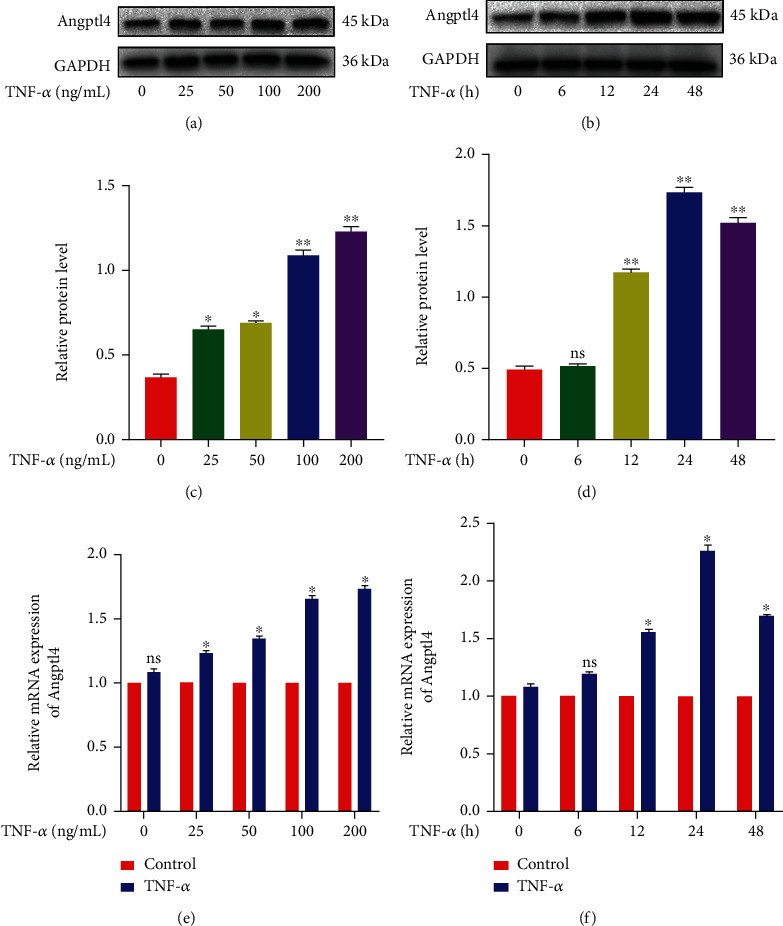
TNF-*α* promoted the expression of angiopoietin-like protein 4 (Angptl4) in chondrocytes. (a, c, e) The chondrocytes were treated with TNF-*α* at 0, 25, 50, 100, and 200 (ng/mL) for 24 hours. The protein and mRNA expression of Angptl4 was determined using Western blotting assay and qRT-PCR, respectively. (b, d, f) The chondrocytes were treated with TNF-*α* (50 ng/mL) for 0, 6, 12, 24, and 48 h. The protein and mRNA expression of Angptl4 was determined using Western blotting and qRT-PCR, respectively. All data were presented as the mean ± SD (*n* = 5); ns: no significance, ^∗^*P* < 0.05, ^∗∗^*P* < 0.01.

Previous data showed that proinflammatory factors such as TNF-*α* fuel the development of OA [36]. To explore the mechanisms of Angptl4 at molecular levels, chondrocytes were treated with TNF-*α* for 24 h to simulate in vitro OA model. Western blot analysis demonstrated that TNF-*α* upregulated the Angptl4 expression in chondrocytes in a dose and time-dependent manner (Figures 2(a)–2(d)). Accordingly, Angptl4 transcription was markedly elevated due to TNF-*α* stimulation (Figures 2(e) and 2(f)). These results were consistent with our *in vivo* findings, indicating that Angptl4 is upregulated in OA.

### 2.3. Angptl4 is Associated with ECM Degradation, Inflammation, and Apoptosis in Chondrocytes

**Figure 3:**
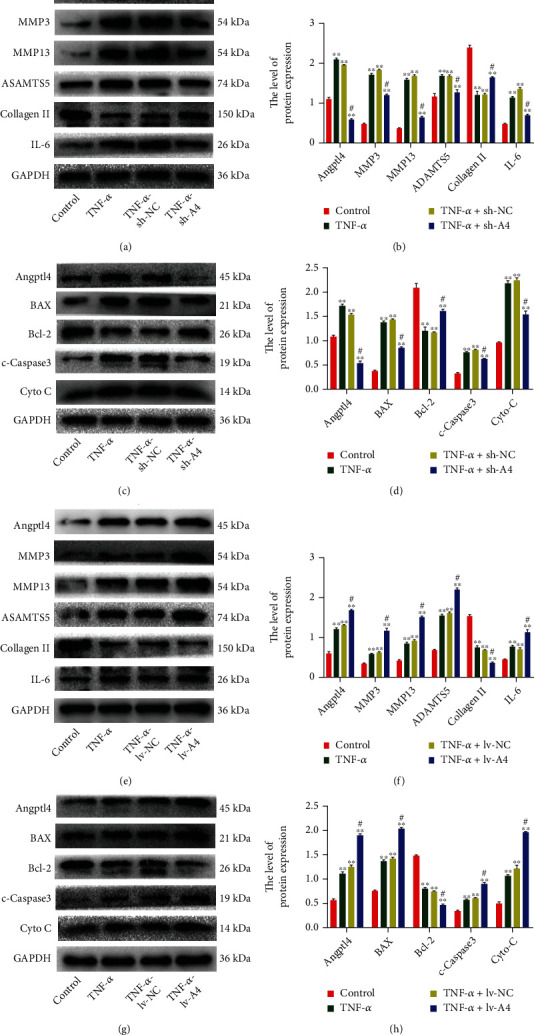
Angptl4 promotes ECM degradation, inflammation, and apoptosis in chondrocytes. The chondrocytes were treated with TNF-*α* (50 ng/mL) except for the control group. (a, b, e, f) Results of Western blotting showing the protein expression level of Angptl4, MMP3, MMP13, ADAMTS5, Collagen II, and IL-6 in chondrocytes treated as above. (c, d, g, h) Results of Western blotting showing the protein expression level of Angptl4, BAX, Bcl-2, C-caspase3, and Cyto-C in chondrocytes treated as above. All data are presented as the mean ± SD (*n* = 5); ^∗∗^*P* < 0.01 vs. control group, ^#^*P* < 0.01 vs. TNF-*α* group.

To better understand the TNF-*α*-mediated Angptl4 functions in OA, lenti-Angptl4 (lv-Angptl4) and lenti-sh-Angptl4 (sh-Angptl4) were used for Angptl4 overexpression and knockdown in chondrocytes, respectively. Western blot assay was performed to measure the transfection efficiency. As expected, there was degradation of ECM in chondrocytes treated with TNF-*α*. Interestingly, inhibiting Angptl4 expression reduced the expression of MMP3, MMP13, and ADAMTS5 and enhanced the expression of Collagen II (Figures 3(a) and 3(b)). In addition, the upregulation of Angptl4 markedly increased the expression of ADAMTS5, MMP3, and MMP13 and promoted the degradation of Collagen II (Figures 3(e) and 3(f)). In addition, TNF-*α* treatment induced apoptosis and release of inflammatory factors, findings that were in sync with results from a previous study [37]. Interestingly, inhibiting Angptl4 expression was shown to reduce the expression of apoptosis-related protein BAX, cleaved-caspase3 (C-caspase3), cytochrome c (Cyto-c), and inflammatory cytokine IL-6 as well as increase in expression of Bcl-2 expression (Figures 3(a)–3(d)). However, the upregulation of Angptl4 exerted conflicting effects on protein expression (Figures 3(e)–3(h)). Moreover, our ELISA and RT-qPCR results demonstrated that knockdown of the Angptl4 expression could reduce the expression of proinflammatory cytokines, IL-6 and IL-1*β*, whereas the Angptl4 overexpression upregulated the expression of proinflammatory cytokines, IL-6 and IL-1*β* (Figure S1. A-D). Together, these results suggested that Angptl4 enhanced the adverse effects of TNF-*α* in chondrocyte degeneration by promoting the catabolism of ECM, apoptosis, and the release of inflammatory cytokines.

### 2.4. Angptl4 Regulates the NF-*κ*B Pathway in TNF-*α*-Induced Chondrocytes

**Figure 4:**
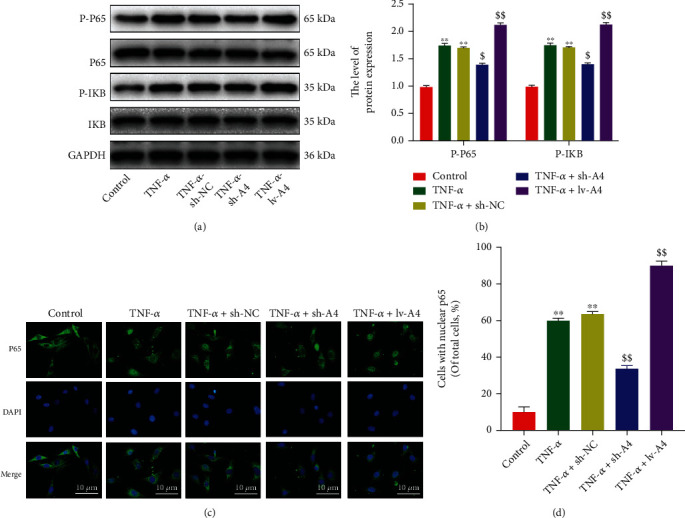
Angptl4 promoted ECM degradation and inflammation through NF-*κ*B activation. The chondrocytes were treated with TNF-*α* (50 ng/mL) except for the control group. (a, b) The phosphorylation level of P65 and I*κ*B*α* in chondrocytes as determined by Western blotting. (c, d) Immunofluorescence staining of P65 in chondrocytes as treated above (bar: 10 *μ*m). All data are presented as the mean ± SD (*n* = 5); ^∗∗^*P* < 0.01 vs. control group, ^$^*P* < 0.05, ^$$^*P* < 0.01 vs. TNF-*α* group.

The highly activated NF-*κ*B signaling pathway has been previously suggested to be associated with OA pathogenesis, and Angptl4 plays a crucial role in regulating the NF-*κ*B signaling pathway [38, 39]. Here, we speculated that Angptl4 might aggravate adverse effects of TNF-*α* by regulating the NF-*κ*B signal transduction in chondrocytes. To test this hypothesis, sh-Angptl4 or lv-Angptl4 were used to upregulate or downregulate Angptl4 protein in chondrocytes stimulated by TNF-*α*. Western blot analysis demonstrated that the Angptl4 silencing decreased TNF-*α*-stimulated phosphorylation of p65 and I*κ*B*α* proteins (Figures 4(a) and 4(b)). In addition, immunofluorescence analysis of p65 showed that elevated Angptl4 expression increased TNF-*α*-induced nuclear translocation of p65 (Figures 4(c) and 4(d)). These results suggested that Angptl4 regulates the NF-*κ*B pathway in TNF-*α*-induced mouse chondrocytes.

### 2.5. Angptl4 Knockdown Reduces TNF-*α*-Induced ECM Degradation and Inflammation in Chondrocytes through the SIRTUIN 1/NF-*κ*B Pathway

**Figure 5:**
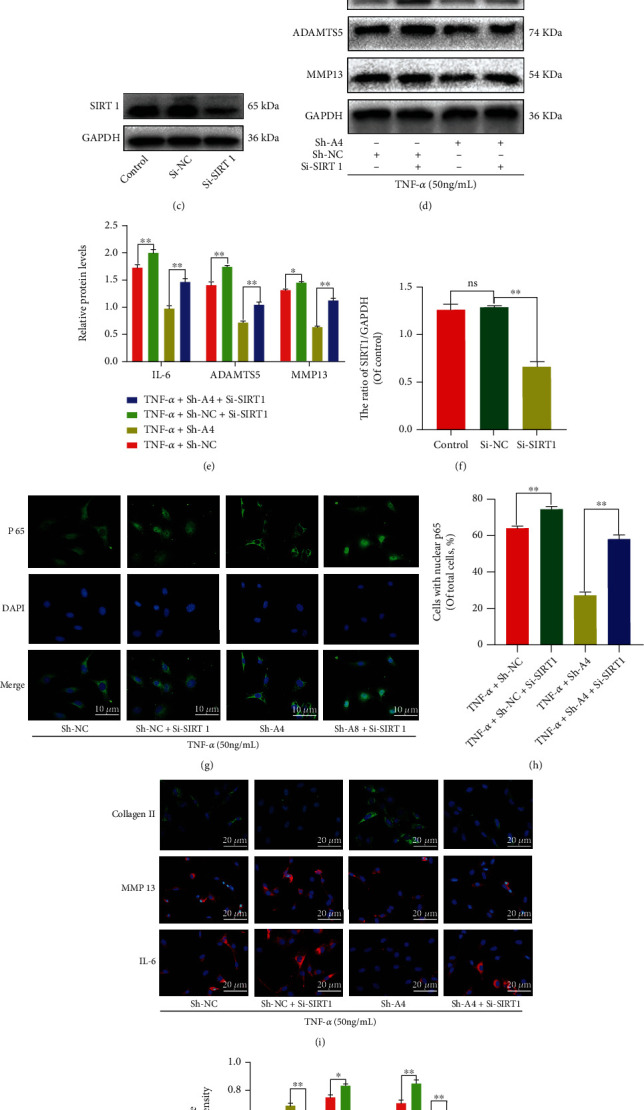
Angptl4 knockdown regulated ECM degradation and inflammation through the SIRTUIN 1/NF-*κ*B pathway. (a, b) Expression level of P65, Lamin B, SIRTUIN 1, and GAPDH in chondrocytes as determined by Western blotting. (c, d) Confirmation of the successful knockdown of SIRTUIN 1 via siRNA. (e, f) The expression level of IL-6, Collagen II, ADAMTS5, MMP3, and MMP13 in chondrocytes as determined with Western blotting. (g, f) Immunofluorescence staining of P65 in chondrocytes after the indicated groups (bar: 10 *μ*m). (i, j) Immunofluorescence staining of Collagen II, MMP13 and IL-6 in chondrocytes as treated above (bar: 20 *μ*m). All data are presented as the mean ± SD (*n* = 5); ns: no significance, ^∗∗^*P* < 0.01.

To assess whether SIRTUIN 1/NF-*κ*B pathway modulates the effects of Angptl4 on ECM degradation and inflammation in TNF-*α*-induced chondrocytes, we isolated nuclear and cytosol proteins from chondrocytes and analyzed the SIRTUIN 1 expression in the cytoplasm and p65 expression in the nucleus via Western blot analysis. Our data showed that the knockdown of Angptl4 reduced the expression of P65 in the nucleus and enhanced the expression of SIRTUIN 1 (Figures 5(a) and 5(b)). These results demonstrated that Angptl4 suppression may inhibit the SIRTUIN 1/NF-*κ*B pathway activation in TNF-*α*-induced mouse chondrocytes.

In addition, to define the role of SIRTUIN 1/NF-*κ*B pathway in regulating Angptl4-mediated ECM and inflammation, we used si-SIRTUIN 1 to suppress SIRTUIN 1 expression in chondrocytes (Figures 5(c) and 5(d)). As shown in Figure 5, the effects of Angptl4 knockdown against TNF-*α*-based ECM degradation and inflammation including reduction of the IL-6, ADAMTS5, MMP3, and MMP13 protein levels, and increasing Collagen II protein levels, were markedly abolished by cotreatment of the chondrocytes with Si-SIRTUIN 1 (Figures 5(e) and 5(f)). In addition, immunofluorescence assay showed that Si-SIRTUIN 1 reversed the protection of Angptl4 knockdown against TNF-*α*, which included reduction of fluorescence intensity of p65 in the nucleus, MMP13 and IL-6, and increasing the fluorescence intensity of Collagen II, findings that were consistent with our Western blot analysis (Figures 5(g) and 5(j)). In summary, our results demonstrated that Angptl4 regulates ECM degradation and inflammation of chondrocytes through the SIRTUIN 1/NF-*κ*B pathway.

### 2.6. Angptl4 Knockdown Reduces TNF-*α*-Induced Apoptosis through the SIRTUIN 1/NF-*κ*B Pathway

**Figure 6:**
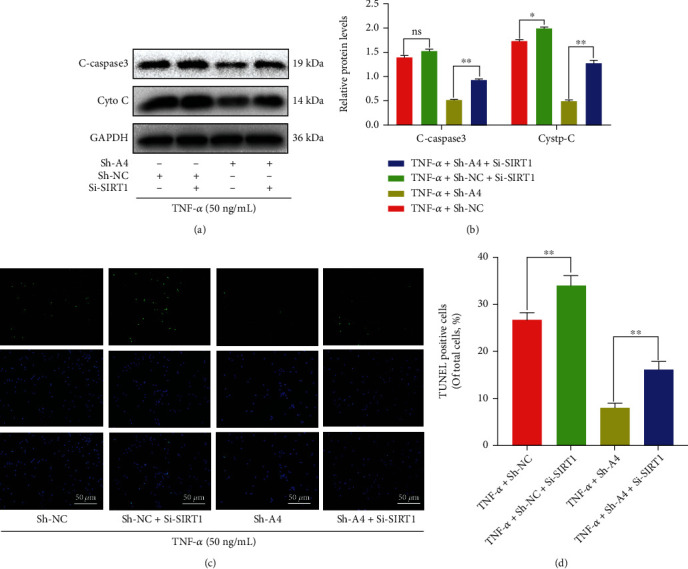
Angptl4 knockdown regulated apoptosis through theIRT1/NF-*κ*B pathway. (a, b) The protein expression level of BAX, Bcl-2, C-caspase3, and Cyto-C in chondrocytes after treatment as determined with Western blotting. (c, d) TUNEL staining assay was conducted on the chondrocytes, as treated above (bar: 50 *μ*m). All data are presented as the mean ± SD (*n* = 5); ns: no significance, ^∗^*P* < 0.05 vs. control group, ^∗∗^*P* < 0.01.

To investigate whether Angptl4 regulates SIRTUIN 1/NF-*κ*B pathway to reduce TNF-induced apoptosis in chondrocytes, we performed Angptl4 knock down and analyzed the phenotypes using Western blot and TUNEL staining assays. As shown in Figure 6, cotreatment of the chondrocytes with Si-SIRTUIN 1 attenuated the effect of Angptl4 knockdown against TNF-*α*-based apoptosis of chondrocytes, which included reduction of apoptosis-related proteins (BAX, Cyto-c, and cleaved caspase-3) and increase of Bcl-2 protein levels (Figures 6(a) and 6(b)). Furthermore, TUNEL staining assay showed that Si-SIRTUIN 1 reversed the protection of Angptl4 knockdown against TNF-*α*, including reduction of the rate of apoptotic cells (Figures 6(c) and 6(d)). These results showed that Angptl4 regulated apoptosis of chondrocytes through the SIRTUIN 1/NF-*κ*B pathway.

### 2.7. Angptl4 Knockdown Ameliorates OA Development in DMM Mouse Model

**Figure 7:**
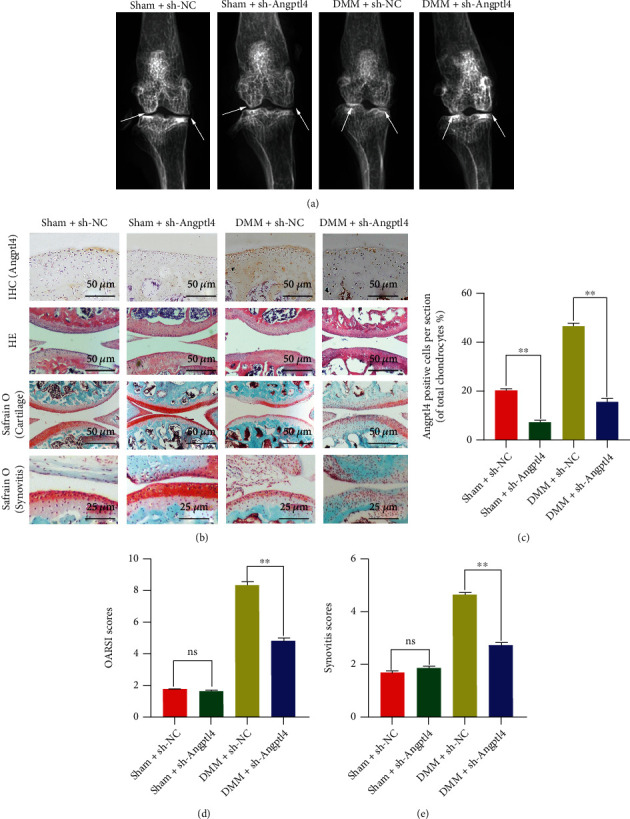
Angptl4 knockdown ameliorated osteoarthritis *in vivo*. Establishment of a mouse model of osteoarthritis surgical destabilization of the medial meniscus (DMM). After 8 weeks, the pathology of OA was assessed by X-ray, H&E staining, and Safranin O staining. (a) Digital X-ray image of mouse knee joints from different experimental groups. (b) Representative immunohistochemistry images of Angptl4, HE, and S-O staining results of the cartilage and synovitis in the four groups at eight weeks postsurgery (bar: 50 or 25 *μ*m). (c) Quantitative analysis of immunohistochemical results of Angptl4. (d, e) The OARIS scores of cartilage and scores of synovitis in the four groups. All data are presented as the mean ± SD (*n* = 10), ns: no significance, ^∗∗^*P* < 0.01.

**Figure 8:**
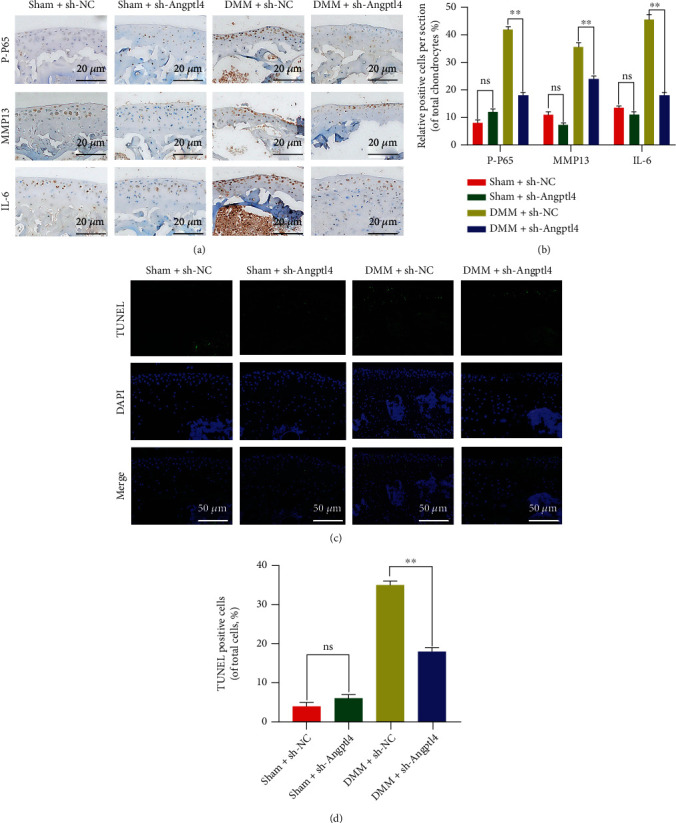
Angptl4 knockdown ameliorated the degradation of ECM, inflammation, and apoptosis by inhibiting NF-*κ*B *in vivo*. (a, b) The expression of p-P65, MMP13, and IL-6 in mouse cartilage as examined by immunohistochemistry (bar: 20 *μ*m). (c, d) Results of the TUNEL staining assay in mouse cartilage (bar: 50 *μ*m). All data are presented as the mean ± SD (*n* = 5); ns: no significance, ^∗∗^*P* < 0.01.

To evaluate therapeutic effect of the knockdown of Angptl4 *in vivo*, lentivirus particle sh-Angptl4 was injected into the knee cavity of mice. Immunohistochemical staining of Angptl4 showed that the expression of Angptl4 was significantly downregulated following the lentivirus injection (Figures 7(b) and 7(c)). Besides, to evaluate the differences in the histological and morphological characteristics, we performed X-ray imaging and tissue staining, including S-O and HE staining. After eight weeks, the DMM + sh-NC mice had narrower joint space and higher cartilage surface density compared with Sham + sh-NC mice. In addition, the DMM + sh-Angptl4 mice had decreased joint space. Besides, there was erosion of articular cartilage surface, decrease in the number of cells, and loss of proteoglycan as well as thickening and proliferation of the synovium in the DMM + sh-NC mice. Compared with the DMM + sh-NC mice, there was complete cartilage surface in the DMM + sh-Angptl4 mice, with thinner synovium and more proteoglycan. Compared with DMM + sh-NC mice, the OARSI and synovitis score of the International Osteoarthritis Research Association markedly decreased in the DMM + sh-Angptl4 mice (Figures 7(a) and 7(e)). Together, our immunohistochemical staining and quantitative analysis data demonstrated that the levels of p-p65, MMP13, and IL-6 in chondrocytes decreased after Angptl4 knockdown (Figures 8(a) and 8(b)). Similarly, tissue TUNEL staining showed that the knockdown of Angptl4 decreased the apoptosis level of chondrocytes (Figures 8(c) and 8(d)). In conclusion, the above results suggested that suppression of Angptl4 can improve OA in mice.

## 3. Discussion

OA is a commonly seen musculoskeletal disorder, which is a major cause of disability globally [40]. OA is a degenerative disease associated with age and involves numerous factors in its pathophysiology, which include imbalance in ECM metabolism, inflammation, and apoptosis [41, 42]. Several studies have shown that the overactivation of the NF-*κ*B signaling pathway leads to progression of several diseases, including degenerative and cardiovascular diseases as well as cancer [43, 44]. Studies have shown that, in acute pancreatitis model, the inflammatory response caused by NF-*κ*B can be effectively inhibited by activation of SIRTUIN 1. This study explored the mechanisms of Angptl4 regulation of NF-*κ*B/SIRTUIN 1 and examined its impact on alleviation of ECM metabolism, apoptosis, and inflammation in TNF-*α*-triggered chondrocytes [16].

The angiopoietin-like protein family is associated with metabolism and inflammation, with a majority being proinflammatory factors [45]. For instance, ANGPTL2 and Angptl4 have proinflammatory effects and play a vital role in regulating inflammation and apoptosis [16, 46–50]. ANGPTL proteins can specifically bind to receptors that activate the NF-*κ*B signal transduction pathway, thus producing inflammatory factors [51]. Although Angptl4 has been studied in diverse disorders, data on the regulatory mechanisms of inflammation and apoptosis remain unclear [52–55]. A previous study showed that Angptl4 silencing is can be a candidate for IVDD [56]. Interestingly, our data showed that the knockdown of Angptl4 can improve ECM metabolism, inflammation, and apoptosis in OA through SIRTUIN 1/NF-*κ*B signal pathway.

It has also been demonstrated that NF-*κ*B signal transduction pathway plays a crucial role in enhancing chondrocyte aging and apoptosis, promoting the activities of matrix-degrading enzymes, and regulating the inflammatory response in OA [57, 58]. Therefore, suppressing the NF-*κ*B signal transduction pathway may potentially inhibit the development of OA. In the inactive form, NF-*κ*B is located in the cytoplasm, which later undergoes phosphorylation and rapid translocation into the nucleus after TNF-*α* treatment [59]. SIRTUIN 1 belongs to the yeast SIR2 (silencing information regulator) mammalian ortholog and the sirtuin family and is associated with modulation of cell survival and transcriptional silencing [11]. SIRTUIN 1 interacts with NF-*κ*B RelA/p65 subunit and also deacetylates RelA/p65 at lysine 310^11^ to suppress transcription, which affect transcription of different apoptosis-related genes such as ARGs, BAX, Bcl-2, Cyto-c, or inflammation-related genes (IL-6 and IL-1*β*) and catabolic genes (MMP3, MMP13, and ADAMTSs) [9]. Therefore, we hypothesized that Angptl4 could alleviate TNF-*α*-induced overactivation of the NF-*κ*B pathway in chondrocytes by modulating SIRTUIN 1 expression.

In our study, we showed that Angptl4 was overexpressed in TNF-*α*-induced OA chondrocytes and OA articular cartilages. Silencing the expression of Angptl4 in chondrocytes decreased ECM deterioration, production of proinflammatory factors, and improvement of apoptosis of TNF-*α*-treated chondrocytes. TNF-*α* exposure enhanced the Angptl4 expression, while Angptl4 overexpression exerted contradictory effects on Angptl4 knockdown in the presence of TNF-*α* treatment. Our results showed that silencing Angptl4 reduced the NF-*κ*B signal transduction pathway while its overexpression promoted the NF-*κ*B signal transduction pathway. In addition, in agreement with our in vitro results, our in vivo data showed the degree of deterioration of articular cartilage and demonstrated that silencing Angptl4 partially delayed OA development.

To evaluate the association between SIRTUIN 1 and NF-*κ*B activation, Si-SIRTUIN 1 was used to knock down the expression of SIRTUIN 1 in chondrocytes. As shown in Figures 5 and 6, Si-SIRTUIN 1 alleviated the inhibition of Sh-A4 on the TNF-*α*-mediated expression of p65 in the nucleus, thus showing the role of SIRTUIN 1 in enhancing Sh-A4 inhibition on TNF-*α*-mediated proinflammatory and proapoptotic effects. These results demonstrated that Angptl4 increased the TNF-*α*-mediated ECM deterioration, apoptosis, and inflammation in chondrocytes through the SIRTUIN 1/NF-*κ*B signal transduction pathway. Consistent with a previous study [21], our study showed that Angptl4 promoted the degeneration of chondrocytes.

Although our study yielded important findings, it was limited by only examining only the intracellular Angptl4 functions in chondrocyte degradation. However, extracellular Angptl4 or the undefined binding receptor may also be participating in OA. Besides, in addition to chondrocytes, OA progression is also associated with fibroblasts such as synoviocytes and synovial macrophages. Thus, there is a need for more studies which explore additional cell types associated with OA. In addition, hypoxia and TNF-*α*-induced chronic inflammation might not be the only factors that promote Angptl4 expression in chondrocytes. Therefore, there is a need for further studies on the role of Angptl4 in OA.

**Figure 9:**
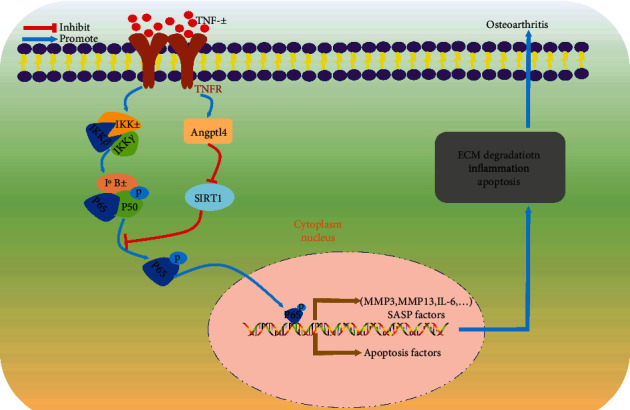
Schematic graph of the role of Angptl4 in TNF-*α*-induced chondrocyte ECM catabolism, inflammation, and apoptosis.

Taken together, this study demonstrated the involvement of Angptl4 in OA development. Angptl4 silencing decreased ECM degradation, apoptosis, and inflammation in chondrocytes by suppressing the SIRTUIN 1/NF-*κ*B signaling pathway (Figure 9). In addition, this study indicated that Angptl4 silencing alleviates OA development in the DMM mouse model. Therefore, Angptl4 is a potential therapeutic target in OA.

## 4. Materials and Methods

### 4.1. Reagents and Antibodies

Recombinant mouse TNF-*α* was acquired from Sigma-Aldrich (654245, St. Louis, MO, USA), while antibodies against Angptl4 were purchased from Sigma-Aldrich (AB10605, St Louis, MO, USA). Antibodies against P65(80979-1-RR), MMP3(18165-1-AP), BAX (50599-2-Ig), Bcl-2(26593-1-AP), cleaved caspase3(19677-1-AP), and Cytochrome c(10993-1-AP) were obtained from Proteintech (Rosemont, IL, USA), while antibodies against C-caspase 3(9661S), P-P65(3039S), and I*κ*B*α*(9242S) were purchased from Cell Signaling Technology (Beverly, MA, USA). Antibodies against GAPDH (ab8245), IL-6 (ab290735), ADAMTS5 (ab41037), Collagen II (ab34712), P-I*κ*B*α*(ab133462), MMP13 (ab39012), SIRTUIN 1 (ab110304), and Lamin B (ab232731) were obtained from Abcam (Cambridge, MA, USA).

### 4.2. Human Cartilage and Chondrocyte Culture

Eight asymptomatic donor animals were collected, and then, OA imaging characteristics were determined from normal articular cartilage of femoral condyle and tibial plateau after autopsy (age, 44–66 years; average, 53.9 years; Kellgren–Lawrence grade, 0 or I; *n* = 8). Total knee arthroplasty was performed in eight cases (age, 45–62 years; average, 56.6 years; Kellgren-Lawrence grades III-IV; *n* = 8). Maximum attention was paid to the safety, anonymity, and confidentiality of the patients' data. After dissecting the human articular cartilage samples into 5 *μ*m sagittal sections, they were embedded in paraffin before histomorphological analyses. The remaining human articular cartilage specimens were then cut into pieces, followed by incubation for 4 h with type II collagenase (2 mg/mL) in the Dulbecco's Modified Eagle Medium (DMEM/F12) at 37°C. Thereafter, chondrocytes were washed in phosphate-buffered saline (PBS), and then, 3.5 × 10^−5^ cells/mL were cultured in DMEM/F12 medium (Gibco, Invitrogen, Grand Island, NY) containing 10% fetal bovine serum (FBS; Hyclone, Thermo Scientific, Logan, UT, USA) and 1% antibiotics (Gibco, Invitrogen, Grand Island, NY), at 37°C and 5% CO_2_. Finally, the chondrocytes at second passage were selected for further analysis.

### 4.3. Isolation and Culture of Chondrocytes

We extracted chondrocytes following a previously described study [60]. Briefly, the mouse knee joint cartilage was cut into small pieces and digested with 0.1% collagenase II (2 mg/mL) for 4 h at 37°C. Subsequently, the tissue pieces were digested and inoculated in the culture flasks. Thereafter, the chondrocytes were grown in DMEM/F12 containing 10% FBS and 1% antibiotics and then incubated at 37°C under 5% CO_2_ for 24 h. Thereafter, the medium was changed, and the second or third generation cells were taken for follow-up experiment and quantify the expression.

### 4.4. Quantitative Real-Time Polymerase Chain Reaction

Trizol reagent (Invitrogen) was used to isolate total cellular RNA from human chondrocytes. The isolated RNA was then used to synthesize cDNA through reverse transcription and then amplified using quantitative real-time polymerase chain reaction (qRT-PCR). The cycle threshold (Ct) values were normalized to the level of GAPDH. The 2^−ΔΔCt^ method was used to quantify the relative expression. Primer sequences used in this study are as follows: for Angptl4, forward 5′-GCCGCACAATAGAACTCCTG-3′, reverse 5′-CAAATTCTCGGTAGGCAGGG-3′; for IL-6, forward 5′-CCGGAGAGGAGACTTCACAG-3′, reverse 5′-TCCACGATTTCCCAGAGAAC-3′; while for IL-1*β*, forward 5′-TGGCAACTGTTCCTG-3′, reverse 5′-GGAAGCAGCCCTTCATCTTT-3′.

### 4.5. Western Blot Analysis

Radioimmunoprecipitation assay buffer containing 1 mM phenylmethanesulfonyl fluoride (PMSF) was used for extraction of total cellular protein from chondrocytes, and then, BCA protein detection kit (Beyotime) was used to analyze the protein content. Subsequently, sodium dodecyl sulfate-polyacrylamide gel electrophoresis (SDS-PAGE) was used to resolve the proteins (30 ng), which were then transferred onto 0.45 *μ*m polyvinylidene fluoride membranes (Bio-Rad, California, USA). The membranes were incubated with 5% skimmed milk (or 5% BSA) for 2 h and then incubated with primary antibody overnight at 4°C. The membranes were washed and then incubated with secondary antibody for 2 h at ambient temperature. The blots were then rinsed three times by TBS, and then, enhanced chemiluminescent plus reagent (Invitrogen, Carlsbad, USA) was used for analysis. Finally, Image Lab 3.0 (Bio-Rad) was used to quantify the protein expression.

### 4.6. Immunohistochemical Analysis

After deparaffinizing and rehydration of the specimens, 3% H_2_O_2_ was used to block the endogenous peroxidase in paraffin-embedded sections. Thereafter, 0.4% trypsin was used to incubate the tissue sections for 30 min for antigen retrieval. Subsequently, 5% bovine serum albumin (BSA) was used to incubate the sections at ambient temperature for 40 min, which were then incubated overnight with primary and horseradish peroxidase-conjugated secondary antibodies at 4°C in succession. In addition, a 3,3′-diaminodbenzidine chromogenic kit (Zhongshan Biotechnology) was used for development. At least three sections of each specimen were analyzed, and positive cells in each section were counted. Finally, Image J software 2.1 (Bethesda, Maryland, USA) was used to quantify positive points.

### 4.7. Immunofluorescence Analysis

The chondrocytes were fixed with 4% paraformaldehyde on a glass slide and the permeabilized with 0.1% Triton X-100 for 15 min. The samples were rinsed using PBS at 37°C and then incubated with 10% goat serum for 1 h. Thereafter, the cells were incubated with primary antibody in a wet chamber at 4°C overnight followed by incubation with Alexa Fluor 488 or Alexa Fluor 594 coupled with the second antibody for 1 hours, then DAPI for 5 min at room temperature. The samples were analyzed under a fluorescence microscope (Olympus, Tokyo, Japan), and Image J 2.1 (Bethesda, Maryland, USA) was used to quantify the fluorescence intensity.

### 4.8. Enzyme-Linked Immunosorbent Assay

After different pretreatments of chondrocytes, we employed enzyme-linked immunosorbent assay (M6000B, ELISA; R&D Systems, Inc., Minneapolis, MN) to examine interleukin (IL)-6 and NO levels secreted by chondrocytes in the cell culture supernatants.

### 4.9. Lentivirus Transduction

Both lenti-sh-Angptl4 and lenti-Angptl4, provided by GeneChem (Shanghai, China), were transfected into cells which were then cultured up to 30%–50% confluence. After 12 h, there were more than 95% alive cells. The medium was replaced, and then, the cells were cultivated for three days prior to further analysis. Western blot assay was performed to assess knockdown efficiency.

### 4.10. siRNA Transfection

si-SIRTUIN 1 was obtained from Invitrogen (Carlsbad, CA, USA). Cells were cultivated in six-well plates until 60%–70% cell confluence. Thereafter, Lipofectamine 2000 siRNA transfection system (Thermo Fisher, UT, USA) was used to transfect 50 nM siRNA or control duplex to the cells before Western blot analysis.

### 4.11. TUNEL Staining

The level of DNA damage was detected by TUNEL staining. First, the chondrocytes were fixed with 4% paraformaldehyde for 1 h, followed by incubation with 0.2% Triton X-100 for 10 min. The cells were then rinsed thrice in PBS. We used in situ Cell Death Detection Kit (Roche, South San Francisco, CA) to stain cells, while DAPI was used to stain the nuclei. Finally, chondrocyte apoptosis was analyzed using Olympus fluorescence microscope (Olympus, Tokyo, Japan).

### 4.12. Animal Model

A total of 60 C57BL/6 mice (10 weeks old, including 30 males and 30 females) were provided by the Animal Center of the Chinese Academy of Sciences (Shanghai, China). As previously mentioned [61], OA mice were established by surgical instability of medial meniscus and then randomly divided into Sham + Sh-NC, Sham+Sh-Angptl4, DMM + Sh-NC, or DMM + Sh-Angptl4 groups. After 0, 15, 30, and 45 days of OA, 10 *μ*L of lentivirus was injected into the joint space through transpatellar tendon approach. The Sham + Sh-NC group or DMM + Sh-NC group received 10 *μ*L Sh-NC. On the other hand, the Sham+Sh-Angptl4 or DMM + Sh-Angptl4 received 10 *μ*L Sh-Angptl4. After eight weeks of OA, the mice in each group were sacrificed for dissection of knee joints which then underwent histological analysis.

### 4.13. X-ray Imaging

Eight weeks following the surgical intervention, all mice were examined through X-rays. To evaluate the joint space, each mouse was subjected to X-ray imaging using the digital X-ray device (Kubtec Model XPERT.8; KUB Technologies) at 160 *μ*A and 50 KV.

### 4.14. Histopathological Analysis

At eight weeks postoperative, the animals were anesthesized by intraperitoneal injection with 2% pentobarbital and then sacrificed for the assessment of knee joints. Subsequently, 4% (*v*/*w*) paraformaldehyde was used to fix all the samples for 24 h, followed by decalcification with 10% (*v*/*w*) ethylenediaminetetraacetic acid. After dehydration, the samples were embedded with paraffin and cut into 5 *μ*m sections. Thereafter, hematoxylin (HE), Alcian blue, and S-O stains were used to stain slides. The Osteoarthritis Research Society International (OARSI) guidelines were adopted to evaluate the medial tibial platform and medial femoral condyle scoring system.

### 4.15. Statistical Analysis

Statistical analyses were carried out in SPSS statistical software program 20.0 (IBM, Armonk, NY, USA). To compare the control and treatment groups, the data were analyzed by one-way analysis of variance (ANOVA), followed by Tukey's test. The nonparametric data (OARSI grading and synovitis scores) were analyzed using the Kruskal–Wallis *H* test. A *P* value of <0.05 was used to indicate statistical significance.

## Data Availability

The data used to support the findings of this study are included within the article.

## Supplementary Materials

Supplementary 1 Supplementary materials: figure S1 and figure legends.

Supplementary 2 Original Western blot: all original Western blot.
