# Supplementary material for: Silencing of Angiopoietin-Like Protein 4 (Angptl4) Decreases Inflammation, Extracellular Matrix Degradation, and Apoptosis in Osteoarthritis via the Sirtuin 1/NF-κB Pathway
Source: Oxid Med Cell Longev. 2022 Aug 27;2022:1135827. doi: 10.1155/2022/1135827 (PMC9442503; doi:10.1155/2022/1135827)
Supplement: Supplementary 1 — Supplementary materials: figure S1 and figure legends. [file 1135827.f1.docx]

**
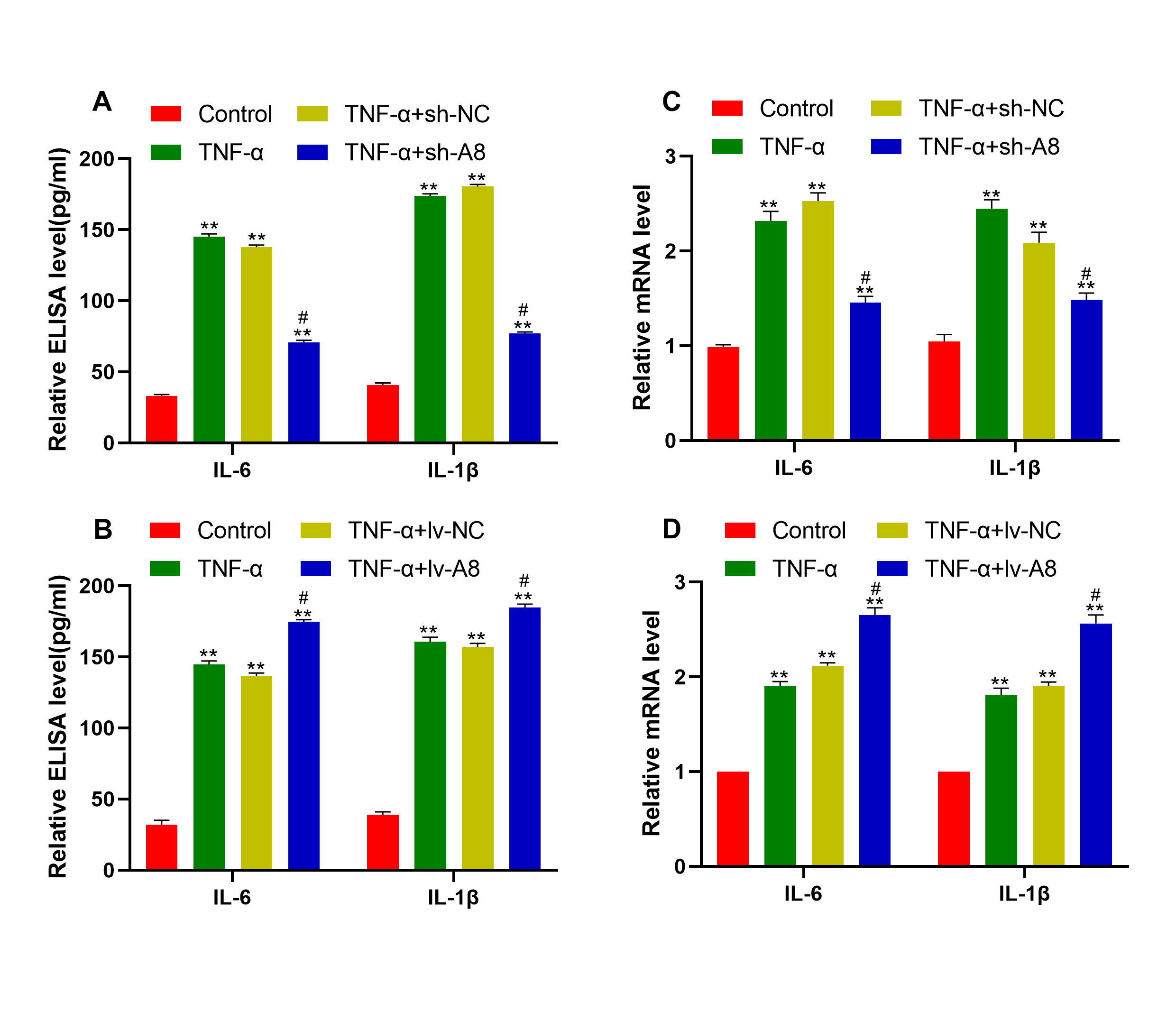
**

**Fig. S1 Angptl4 promotes inflammation in chondrocytes. The chondrocytes were treated with TNF‐α (50 ng/mL) except for the Control group. (A，B)** ELISA analysis showed the level of IL-6 and IL-1βin culture medium supernatant of chondrocytes treated as above. **(C, D)** qRT‐PCR analysis showed the mRNA level of IL-6 and IL-1βin chondrocytes treated as above. All data were indicated as mean ± SD (n =3); **P < 0.01 vs Control group, #P < 0.05vs TNF-α group.
