# Supplementary material for: Silencing of Angiopoietin-Like Protein 4 (Angptl4) Decreases Inflammation, Extracellular Matrix Degradation, and Apoptosis in Osteoarthritis via the Sirtuin 1/NF-κB Pathway
Source: Oxid Med Cell Longev. 2022 Aug 27;2022:1135827. doi: 10.1155/2022/1135827 (PMC9442503; doi:10.1155/2022/1135827)
Supplement: Supplementary 2 — Original Western blot: all original Western blot. [file 1135827.f2.docx]

https://www.jianguoyun.com/p/DYPGiX0Qu4_lChiQ_MwEIAA
